# Thyroid Gland Disease as a Comorbid Condition in COPD

**DOI:** 10.1155/2021/7479992

**Published:** 2021-10-29

**Authors:** Aziz Gumus, Neslihan Ozcelik, Bilge Yilmaz Kara, Songul Ozyurt, Unal Sahin

**Affiliations:** Recep Tayyip Erdogan University, Faculty of Medicine, Department of Chest Disease, Rize, Turkey

## Abstract

**Introduction:**

Chronic obstructive pulmonary disease (COPD) is one of the most common causes of morbidity and mortality worldwide. The disease is characterized by progressive airway inflammation, which not only affects the airways but also has systemic effects that are associated with comorbidities. Although comorbid conditions such as hypertension and coronary artery disease are very well-known in COPD patients, diseases of the thyroid gland have not been sufficiently studied. Therefore, thyroid diseases are not considered among the comorbid conditions of COPD. The purpose of this study was to determine the thyroid gland disease (TGD) prevalence in COPD and associated factors. *Materials and Method*. The study included 309 (297 (96%) male) patients. The patients were subjected to spirometry and thyroid function tests (TFT) in the stable period. The thyroid gland disease they were diagnosed with was recorded after face-to-face meetings and examining their files.

**Results:**

The mean age of the patients who were included in the study was 65.9 ± 9.8 (40-90). Thyroid disease was determined in 68 (22%) individuals. There were hypothyroidism in 7 (2%), euthyroidism in 45 (15%), and hyperthyroidism in 16 (%5) patients. No relationship was found between the severity of airflow limitation and the prevalence of TGD.

**Conclusion:**

Thyroid abnormalities are commonly observed in COPD. The most frequently encountered TGDs are euthyroid multinodular goiter, euthyroid sick syndrome (ESS), and toxic multinodular goiter.

## 1. Introduction

Chronic obstructive pulmonary disease is one of the most significant causes of mortality and morbidity worldwide especially among former of current smokers over the age of 40 [1]. The World Health Organization projects that it will be the third most significant cause of mortality and morbidity by the year 2030 [2]. COPD is accompanied by other diseases that are expressed as comorbidities and significantly affect its prognosis. While some of these emerge independently of COPD, some of them are interrelated due to common risk factors such as smoking and advanced age [3]. As the presence of comorbidities significantly affects the management of COPD, the GOLD (Global Initiative for Chronic Obstructive Lung Disease) guidelines prominently emphasize the importance of comorbidities [4] which not only disrupt quality of life but also increase mortality in advanced-stage COPD patients [5]. The most frequently encountered comorbidities are cardiovascular diseases, lung cancer, obstructive sleep apnea syndrome, diabetes mellitus, metabolic syndrome, and osteoporosis. Anxiety, depression, skeletal muscle myopathies, and mental disorders are also seen as comorbid conditions [6].

Thyroid hormones play an important role in the regulation of the metabolism. Changes in the serum thyroid hormone levels arise in many systemic diseases. COPD leads to thyroid dysfunctions not only through systemic inflammation but also by affecting the endocrine homeostasis [7]. In relation to blood gas abnormalities that develop during the course of COPD, thyroid function disorders such as hypothyroidism, hyperthyroidism, and nonthyroidal illness syndrome emerge [8]. A limited number of studies have been conducted on TGD in COPD. They were found to be more prevalent among female patients than males [9]. The purpose of our study was to investigate the prevalence of TGD as a comorbid condition in COPD and related factors.

## 2. Materials and Method

Patients who were being followed up with the diagnosis of COPD and visited the outpatient clinic in a 1-year period between June 2019 and May 2019 were included in the study. The initial diagnosis of TGD was made by conducting anamnesis, physical examination, and file examination procedures. Drug use that may have an effect on thyroid functions (corticosteroids, amiodarone, iodinated contrast agents) was questioned. Three patients using corticosteroids were excluded from the study. In the stable period, all patients were subjected to 6-minute walking distance test, spirometric measurements of lung volumes, arterial blood gas analyses, TFT, and biochemical measurements including complete blood count. Their smoking history and other comorbid conditions were recorded. GOLD staging was performed according to the GOLD 2020 criteria. Those in whom anomalies were determined by thyroid gland examination and TFT were referred to the endocrinology department for further examination. The ethics committee approval was obtained from Recep Tayyip Erdogan University Clinical Research Ethics Committee (Ethics Committee Approval date: 08/05/2019 and ID: 2019/79).

### 2.1. Spirometric Measurement

Lung volume and flow measurements (forced expiratory volume in 1 second (FEV1), forced vital capacity (FVC), FEV1/FVC, forced mid-expiratory flow (FEF25-75), and peak expiratory flow (PEF)) were obtained by using a Flowhandy ZAN 100 USB Pulmonary Spirometer device (nSpire Health, Inc., Germany). The measurements were carried out with at least three replicates when the patient was in a straight sitting position. The highest value was accepted as the test result. The results were calculated by the help of a computer programme, based on the demographic data of the patients by using reference values for each age group.

### 2.2. Thyroid Function Test Values and Definitions of Hyper/Hypothyroidism

Hyperthyroidism was defined as serum thyroid stimulating hormone (TSH) concentration below the lower limit of the reference range (0.35-4.94 mIU/ml) and free tri-iodothyronine (fT3) and/or free thyroxine (fT4) values above the reference values (1.71-3.71 pg/ml for fT3 and 0.7-1.48 ng/dl for fT4). Hypothyroidism was defined as the presence of an elevated TSH with low serum levels of fT3 and/or fT4.

### 2.3. Statistical Analysis

Statistical analyses were carried out by using the IBM-SPSS software (SPSS version 22; SPSS Inc., Chicago, IL, USA). The Kolmogorov-Smirnov test was used to test the normal distribution of variables. Parametric and nonparametric statistical methods (Student's *t*-test for paired and unpaired data, *Mann*–Whitney *U*-test for unpaired data) were used, when indicated, in the primary analyses of the data. The continuous variables are presented as mean ± standard deviation. *p* < 0.05 was accepted as statistically significant.

## 3. Results

Among the 309 patients who were included, 297 (96%) were males, and 12 (4%) were females. The mean age of the patients was 65.9 ± 9.8 (40-90). Demographic characteristics, biochemical parameters, and comorbid conditions of the patients are shown in Table 1.

Thyroid dysfunction was found in 68 (22%) patients. The general characteristics of the patients with and without TGD are shown in Table 2.

**Figure 1:**
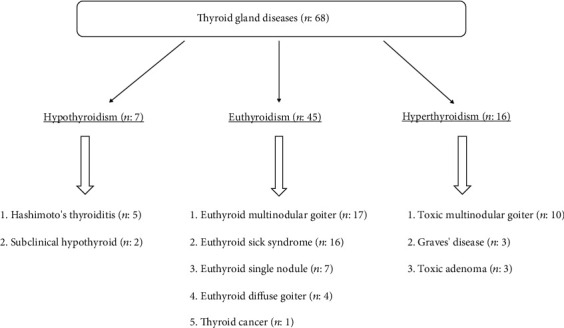
Number of patients in the subgroups of thyroid gland disease.

**Figure 2:**
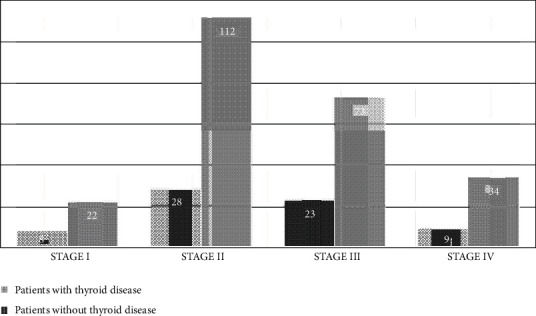
TGD prevalence in COPD patients based on GOLD staging.

When the 68 individuals with TGD were examined in detail, hypothyroidism in 7 (2%), euthyroidism in 45 (15%), and hyperthyroidism in 16 (%5) patients were determined (Figure 1). No relationship was found between the severity of COPD and the prevalence of TGD (Figure 2).

**Figure 3:**
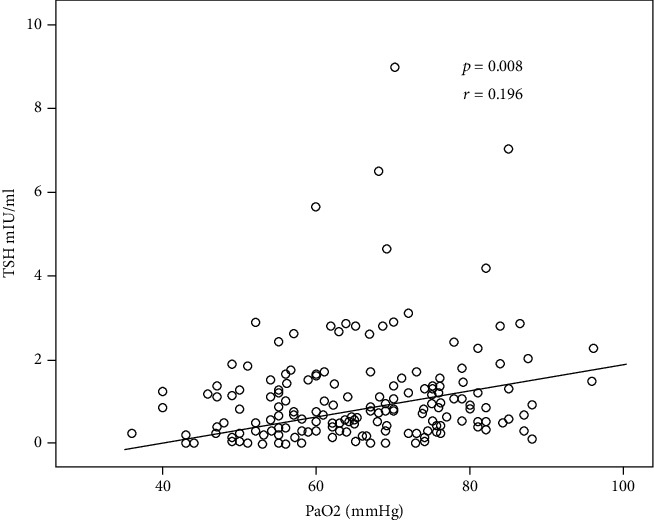
Positive correlation between TSH and PaO2.

The relationship between the TFT and the arterial blood gasses was examined by Pearson's correlation analysis. There was a positive relationship between TSH and PaO2 (*p* = 0.008, *r* = 0.196) and between TSH and oxygen saturation (*p* = 0.027, *r* = 0.164). Similarly, there were also significant, but weak, positive correlations between fT3 and PaO2 (*p* = 0.019, *r* = 0.213) and between fT3 and oxygen saturation (*p* = 0.018, *r* = 0.216). No significant relationship was found between fT4 and arterial blood gasses. Moreover, a significant relationship could not be detected between the PFT and TFT. The scatter plot graph demonstrating a positive correlation between TSH and PaO2 is shown in Figure 3.

The relationship between systemic inflammatory markers and TFT was investigated. A significant negative correlation was found between CRP and TSH and fT3 (*p* < 0.001, *r*: -0.382 and *p*: 0.001, *r*: -0.316, respectively). Similarly, a significant negative correlation was observed between leukocyte counts and TSH and fT3 values (*p* < 0.001, *r*: -0.291 and *p*: 0.003, *r*: -0.273, respectively).

## 4. Discussion

In this study, the prevalence of TGD was found to be as high as 22% among COPD patients with a mean age of 65.9 ± 9.8. The most frequent thyroid gland diseases were those with euthyroidism, followed by hyperthyroidism and hypothyroidism, respectively. When the patients were assessed in terms of the subgroups of TGD, the most frequent diseases were euthyroid multinodular goiter and ESS. We also did not find a difference in the prevalence of TGD based on GOLD staging.

Former studies have emphasized that thyroid diseases are more prevalent among female patients [10]. However, the number of the female patients in our study was very low. In developing countries like Turkey, the higher rate of smoking and COPD among males is an expected situation [11]. In our study, the prevalence of TGD was found as 25% (3/12) in the female patients and 22% (65/297) in the male patients. No significant difference was found between the male and female patients in the prevalence of TGD. It would be more accurate to state that our results reflect the prevalence of TGD in male COPD patients. In a large cross-sectional study which investigated comorbid conditions in COPD patients, García-Olmos et al. determined the prevalence of TGD as 10.9% in male and 24.6% in female patients [9]. While the results was similar in the female patients, the rate among the male patients was higher in our study.

Chaudhary et al. determined TGD in 25.1% of 171 COPD patients. All of these patients consisted of patients with hypothyroidism, and hyperthyroidism was not encountered. In terms of sex, the prevalence of TGD was found as 28.8% in female patients and 23.8% in male patients [8]. However, the difference was not statistically significant. Singh et al. identified thyroid disorders in 130 (64.6%) of 201 COPD patients [12]. Among these, 119 (59.2%) had hypothyroidism, and 11 (5.4%) had hyperthyroidism. In a recent study, Verma et al. [13] determined thyroid disorders in 45 (37.2%) of 121 COPD patients. Eleven subjects were classified as having subclinical hypothyroidism and 33 subjects as having hypothyroidism. Hyperthyroidism was detected in only one patient. While hypothyroidism was dominantly prevalent in all 3 studies, it was reported as the least frequently encountered disease group in our study. The reason for this clear difference may be the detailed examination of specific thyroid diseases in our study. We did not only look at the functional disorders of thyroid but also examined anatomic disorders such as diffuse goiter and thyroid nodules in detail.

In our study, we found the prevalence of ESS as 5% (16/309). While ESS is shown in approximately 70% of the acute exacerbations of COPD, it is detected in about 14-20% of the stable COPD cases [14]. The main mechanism in the development of ESS in stable COPD patients is the reduction in the peripheral transformation of fT4 into the active form of fT3 due to hypoxemia. In ESS, fT3 decreases while TSH may be normal or low. In this study, we showed a positive relationship between PaO2 and fT3 and TSH. As PaO2 decreased, fT3 and TSH also decreased. From the analysis presented here, we can conclude that, in stable COPD patients, hypoxemia contributes to ESS development. In our study, there was no significant relationship between COPD GOLD stages and the prevalence of TGD. Amardeepak Toppo et al. [15] showed that TFT results were lower in COPD patients in comparison with the healthy control group. Nevertheless, they have reported that there was no significant difference in TFT based on COPD stages.

One of the most important results in this study was the demonstration of a negative relationship between inflammatory markers and thyroid function tests. A negative correlation was found between CRP and TSH and fT3. There is a widespread perception that increased inflammation in COPD causes a decrease in TSH level by affecting the pituitary gland and also reduces the formation of fT3 from fT4. Kar et al. [16] reported a significant negative correlation between CRP and TSH (*r* = −0.108, *p* < 0.05), although in a study which included 190 subjects, Lee et al. declared that there was no significant relationship between CRP and TSH [17].

Consequently, both functional and anatomical TGD are comorbid conditions that is frequently encountered in COPD. In addition, ongoing inflammation and hypoxemia in patients with COPD affect thyroid functions. Euthyroid multinodular goiter and ESS are the most frequently encountered subgroups. Because COPD presents with symptoms such as shortness of breath, weakness, loss of appetite, and weight loss, the clinical signs of hyperthyroidism and/or hypothyroidism may be masked, and this can lead to difficulties in diagnosis. As a consequence, delayed diagnosis of TGD will lead to unwanted outcomes in COPD patients; that is because both hypothyroidism and hyperthyroidism affect the respiratory system negatively through different mechanisms. Careful examination of the thyroid gland and measuring TFT at certain intervals while following up COPD patients will contribute to early detection of TGD.

## Figures and Tables

**Table 1:** Demographic characteristics, biochemical parameters, and comorbid conditions of the patients.

Parameters	
Age (years) (mean ± sd)	66 ± 10
Gender, *n* (%)	
Female	12 (4)
Male	297 (96)
Serum biochemical parameters and complete blood count values (mean ± sd)	
Hemoglobin (gr/dl)	14.4 ± 1.7
Hematocrit (%)	43 ± 5
RDW (%)	13.7 ± 2.8
MCV (fl)	86 ± 4
Leukocyte (/ml)	8499 ± 3486
Thrombocyte (/ml)	170724 ± 57793
Glucose (mg/dl)	110 ± 40
Urea (mg/dl)	39 ± 15
Creatinine (mg/dl)	0.91 ± 0.26
CRP (mg/dl)	8.8 ± 2.3
Cholesterol (mg/dl)	189 ± 40
Triglyceride (mg/dl)	130 ± 36
Comorbidities, *n* (%)	
HT	127 (41)
DM	43 (14)
IHD	57 (18)
CHF	18 (6)
CRF	15 (5)
Cancer	45 (15)
CVD	9 (3)
Anxiety	18 (6)
Obesity	4 (1)
Anemia	16 (5)

mean ± sd: mean ± standard deviation; *n*: number; %: percentage; RDW: red cell distribution width; MCV: mean corpuscular volume; CRP: C-reactive protein; HT: hypertension; DM: diabetes mellitus; IHD: ischemic heart disease; CHF: congestive heart failure; CRF: chronic renal failure; CVD: cerebrovascular disease.

**Table 2:** Demographic characteristics of the patients with and without TGD.

	Patients with TGD (*n*: 68)	Patients without TGD (*n*: 241)	*p*
Age (years) (mean ± sd)	65 ± 11	66 ± 9	0.424^∗^
Gender (f/m)	3/65	9/234	0.527
BMI (kg/m^2^)	26.2 ± 5.4	25.8 ± 4.8	0.645^∗^
Smoking (pack-years)	46 ± 28	47 ± 25	0.741^∗^
FEV1 (% predicted)	51 ± 18	47 ± 16	0.184^∗^
FVC (% predicted)	67 ± 20	63 ± 19	0.194^∗∗^
FEV1/FVC	59 ± 10	58 ± 9	0.553^∗∗^
PEF (% predicted)	45 ± 20	41 ± 14	0.163^∗^
FEF25-75 (% predicted)	29 ± 14	27 ± 13	0.223^∗∗^
pH	7.41 ± 0.04	7.40 ± 0.05	0.478^∗∗^
PaO2 (mmHg)	66 ± 13	65 ± 12	0.234^∗^
PaCO2 (mmHg)	40 ± 6	41 ± 7	0.550^∗^
sO2 (%)	93 ± 4	92 ± 5	0.179^∗∗^
6MWD (meters)	397 ± 51	395 ± 74	0.966^∗^

*n*: number; f: female; m: male; BMI: body mass index; mean ± sd: mean ± standard deviation; FEV1: forced expiratory volume in 1 second; FVC: forced vital capacity; PEF: peak expiratory flow; FEF _25-75_: forced midexpiratory flow; PaO2: arterial partial pressure of oxygen; PaCO2: arterial partial pressure of carbon dioxide; 6MWD: 6-minute walking distance. ^∗^Student *t*-test conducted. ^∗∗^Mann-Whitney *U*-test conducted.

## Data Availability

Data are available on request.
